# Becoming a Non-native Chinese Language Teacher: An Identity Triangle Model Analysis

**DOI:** 10.1007/s40841-023-00281-8

**Published:** 2023-03-21

**Authors:** Danping Wang, Claudia Mason

**Affiliations:** 1 School of Cultures, Languages and Linguistics, The University of Auckland, Auckland, New Zealand; 2 Speech and Language Pathology, Faculty of Science, The University of Canterbury, Christchurch, New Zealand

**Keywords:** Teacher identity, Non-native speaker teacher, Identity triangle model, Case study, Mandarin Chinese

## Abstract

This study utilises the Identity Triangle Model (Dugas in Teach Dev 25(3):243–262, 2021, 10.1080/13664530.2021.1874500) to examine the experiences of one particular novice non-native Mandarin Chinese teacher at a university in New Zealand. A case study design was employed to track the identity negotiations of this European non-native Chinese speaker during 12 weeks of her first semester of teaching. Analysis of the data revealed nine subcategories within the psychological, behavioural, and relational domains according to the Identity Triangle Model. The findings suggest that this new non-native speaker teacher viewed her as an accidental teacher, exploring a teaching career without a strong instrumentalist aspiration or a clear career path in language teaching. Instead, she was more motivated by a desire for personal growth and the opportunity to reinvent themselves in a new cultural context. The results of this study offer theoretical implications for the adoption of a unified framework in future research on the identity of first-time language teachers, and practical implications for developing sustainable strategies aimed at recruiting and retaining non-native speaker teachers in foreign language education.

## Introduction

Since World War II, the shortage of foreign language teachers in Anglophone countries has been a longstanding issue that remains unresolved to this day (Swanson & Mason, 2018). While some languages, such as German and Italian, struggle with a decline in student enrolment and teacher supply (Lanvers et al., 2021), other languages, such as Chinese, face difficulties in balancing the abundance of native-speaker teachers (NSTs) and the severe shortage of non-native speaker teachers (NNSTs). The latter have the potential to serve as role models for learners, having learned the additional language themselves typically through formal education (Llurda & Calvet-Terré, 2022). The shortage of NNSTs in Chinese language teaching presents a particularly challenging issue due to the linguistic difficulties (complex writing system and phonemic tones, among other factors) that learners face in becoming proficient in this language (Moser, 1991; Zhang & Wang, 2017). As a result, most Chinese language teachers in Anglophone countries are native speakers born and trained in Greater China. In Australia, over 90% of Chinese language teachers in schools are “ethnic Chinese, most born on the mainland [of China]” (Orton, 2011, p. 153). Similarly, in New Zealand, roughly the same percentage of secondary school Chinese language teachers are native speakers, despite Chinese being the quickest growing language programme in the last decade (Wang, 2021). A similar situation can be observed across all educational levels in other Anglophone countries such as the United States (Yue, 2017), the United Kingdom (Yang, 2019), and Canada (Doherty, 2021).

The lack of diverse representation of race, culture, values, and educational backgrounds (Egalite et al., 2015) has made Chinese language teachers an essentially monocultural group, and their teaching inevitably suffers from insufficient intercultural competence in understanding local student diversity and maintaining their learning interest (see e.g., Moloney, 2013; Liao et al., 2017). Based on a national survey in Australia, Orton (2016) reported a uniform 95% dropout rate for non-ethnic Chinese students who were learning Chinese as a foreign language as soon as language learning was no longer required. Due to the high dropout rate of these students, educators in Australia have lamented that Chinese language education has become “overwhelmingly a matter of Chinese [teachers] teaching Chinese to Chinese” (Scrimgeour, 2014, p. 154), largely failing to attract and engage students whose learning needs are more diverse yet would benefit in many ways from continuing to study to higher levels of proficiency. The situation in New Zealand’s Chinese programmes is equally worrying (Kennedy, 2020).

Over the past three decades, there has been a substantial increase in the number of studies conducted on NNSTs. Plenty of studies have outlined the advantages and disadvantages of NSTs and NNSTs in the field of English language education. In general, NNSTs are often perceived as possessing the advantages of using their first languages in explaining complex cognitive concepts, building rapport with students, and sharing their language learning experiences with students (Calafato, 2019; Thompson & Fioramonte, 2012). Meanwhile, research also reveals that NNSTs are still often perceived as inferior to NSTs, for example, in pronunciation and accent, grammar accuracy, vocabulary and cultural knowledge, as well as their credibility in the language education market (Moussu & Llurda, 2008; Reves & Medgyes, 1994).

Research on NNSTs of languages other than English is scarce (Llurda & Calvet-Terré, 2022). Of the few studies on NNSTs of Chinese (Liu & Wang, 2018; Zhang & Zhang, 2018), much of the focus is on how these teachers have succeeded in learning the language and achieving native-like fluency against all odds. According to Ushioda (2017), this emphasis on native-speakerism and proficiency-based standards often leads to an unfair comparison of NNSTs to NSTs, with NSTs often enjoying a competitive advantage in employment. However, this binary approach overlooks the unique and diverse experiences, identities, and potential of NNSTs in language teaching. There is a significant gap in understanding the crucial transitional phase where a language learner transforms into a teacher, particularly for those who are not yet advanced speakers of the target language. Furthermore, existing models of teacher identity research focus primarily on the English language teaching context globally, meaning that there is a lack of knowledge about the lives, changing identities, and “alternate realities” of NNSTs in Chinese language teaching, where they are in short supply and then face high rates of attrition.

## Literature Review

### The Identity Triangle Model

This study takes a holistic approach to teacher identity, viewing it as an ongoing process of negotiations and self-production towards an integrated and coherent understanding of the self and its place within the socio-cultural context. To follow this whole-person approach, this study will adopt a relatively new conceptual framework in teacher education.

Dugas’ (2021) Identity Triangle Model (ITM), based on interviews with nearly 60 first-year teachers in an urban U.S. context, allows researchers to integrate multiple domains of teacher identity into a unified framework that allows for a more holistic understanding of teachers as humans. This model is based on the idea of connecting teachers’ work and lives to understand their thinking and actions as complete individuals. According to Dugas (2021), “the function of teacher identity is to integrate into a coherent whole an individual’s various experiences as a teacher, not only with one another but also with their other life experiences outside of the teaching context” (p. 245). Under the ITM framework, teacher identity research is no longer restricted to defining identity as an internal psychological phenomenon or an external behavioural or interactional process. Instead, it is viewed as a continuous construction of the life narrative that a person tells themselves and others about themselves.

The Identity Triangle Model consists of three domains: the psychological, behavioural, and interactional. The psychological domain refers to the life narrative or life story (McAdams, 2001) that is continuously (re)constructed and (re)told to oneself. It is influenced by one’s emotions, beliefs, values, and dispositions, but it centres on life narrative construction, as it is through this process that an individual makes sense of themselves as a coherent “I.” The behavioural domain indicates the multiple roles that teachers take on and their concrete actions, such as “instructing classes, managing misbehaviours, building relationships with students, interacting with colleagues and administrators, and so forth” (Dugas, 2021, p. 252). It seeks to de-centre the narrow focus of teacher education on training skills for good and effective competencies in the classroom (Mockler, 2011). The relational domain is about achieving congruence between one’s inner life narrative and how one is positioned by others. It is particularly relevant for novice teachers in the process of transitioning from language learner to teacher (Farrell, 2003; Harrington & Sacks, 2006). These domains are viewed as coequal and interdependent aspects of a person’s identity, connected by the alignment and contradiction of the interactions among each of the three domains.

The Identity Triangle Model challenges the long-standing focus on teacher identity through the lens of classroom outcomes and teacher competency (Mockler, 2011). It is clear that becoming a teacher involves not only professional development, but also a multi-layered personal and social experience that requires a unified framework to integrate these aspects and understand teachers as whole individuals (Alsup, 2006; Pennington & Richards, 2016). However, despite the widespread acceptance and extensive research on this whole-person approach in the context of English language teaching, little attention has been given to Chinese language teachers and their experiences in the three domains of the ITM. Their stories and challenges remain largely unexplored.

### Language Teacher Identity Research

There have been numerous studies published on NNST identity in English language teaching (Llurda & Calvet-Terré, 2022; Moussu & Llurda, 2008). In the context of teaching languages other than English, existing research shows that some studies have similar findings to those on NNSTs of English, while others diverge in many aspects (Thompson & Fioramonte, 2012). One significant finding is that NNSTs tend to highlight a coherent “I” in the process of becoming a teacher. In the following paragraphs, we will demonstrate how NNSTs are not necessarily preoccupied with being accepted by native-speaker communities of the target language or establishing a long-term career in language teaching. Instead, they emphasise a more holistic experience of teaching a language that they learned out of personal interest rather than mandatory education for economic gain.

Focusing on three NNSTs of Spanish in the United States, Thompson and Fioramonte (2012) found that NNSTs did not view themselves as language experts or authorities with native-speaker accuracy. They were confident in their teaching skills and ability to engage and motivate students to learn using locally relevant strategies. Unlike NNSTs of English, they did not show anxiety about making mistakes or having incorrect pronunciation when teaching. They believed that “making mistakes was acceptable” (p. 571) and expected their students to be understanding of their NNST identity. In another study on German as a foreign language, Ghanem (2015) found that NNSTs tended to emphasise their individuality and unique experiences as individuals. The study found that although NNSTs tended to perceive a lack of authority and confidence in the target language, their teaching performance in the classroom was not significantly different from that of their native speaker peers. Furthermore, in Japanese language teaching, Armour (2004) analysed the life story of an Australian Japanese language teacher. The study found that the teacher discovered her sense of “Westernness” when traveling to Japan and yet had “no desire to be less than [her]self” (p. 19). This corresponds to Bamberg and Georgakopoulou’s (2008) understanding that through the constant adjustment of one’s narrative over time, a sense of self-sameness or coherence is created and maintained. Instead of attempting to change or assimilate themselves into a different way of being, they view their professional experiences as an integral part of all valuable experiences that enrich their life stories.

Nevertheless, research on NNSTs of Chinese shows their professional identities are often benchmarked against native Chinese teachers, whereas their whole-person multi-layered social life experience is largely ignored. According to Zhang and Zhang (2018), in Chinese language teaching, NNST identity lacks the subjectivity needed for constructing the foundations of professional identity from the bottom up. Hence, a detailed and longitudinal description of NNSTs’ lived experience is needed but remains understudied (Duff et al., 2013). A lack of role models as reference points may deter any budding Chinese learner curious about becoming a teacher, as they play a crucial role in showcasing the viability and value of the learning journey to students (Doherty, 2021; Liu, 2020). However, due to the perceived linguistic challenge in Chinese learning, most existing publications on NNST identity have focused narrowly on teachers’ language learning strategies and achievements in overcoming their linguistic disadvantages compared with NSTs (Liu & Wang, 2018). In another study, Zhang and Wang (2017) called for a more inclusive and supportive professional environment for novice NNSTs and learners of Chinese to explore the possibilities of becoming teachers despite their limited or developing Chinese language skills. When it comes to achieving a high level of proficiency, language learners usually find the goal of native-level proficiency too remote, particularly in Chinese, which discourages even the most capable learners from even picturing themselves as teachers of that language. As can be seen, the literature on NNST identity in Chinese language teaching has been permeated with native-speaker bias, resulting in an instrumentalist perspective that says one must become highly proficient in the language in order to teach it (Ushioda, 2017).

## The Study

### A Case Study

The study addressed the research question: How does a novice NNST of Chinese construct and negotiate her professional identity during her first teaching experience? Using a case study design, this study provides a thick description and in-depth analysis of a concrete example (one NNST teacher) in a specific context (a university Chinese programme in New Zealand) (Duff, 2018). While case study focuses on individuals and processes in social context, humanistic narrative inquiry is often integrated within studies to let participants (i.e., cases) speak for themselves. The narrative approach emphasises personal voice, identity, agency, and lived experiences that are central to understanding language teachers’ inner world (Bell, 2002), and has become a tradition in teacher identity research (Barkhuizen, 2019).

The case study described in the remainder of this article was conducted in a Chinese language programme in a New Zealand university. Consistent with international trends referred to in the “Introduction” section, interest in learning languages other than English across all educational sectors in New Zealand has been in continuous decline over the past decade (Wang & Chik, 2022). In addition to decreasing demand from learners, becoming a language teacher may not be perceived to be a stable and attractive job by university graduates in the Anglophone West (Swanson & Mason, 2018), including New Zealand. Given the severe shortage of NNSTs in Chinese language teaching, for this study it was not possible to find a group of NNSTs starting their teaching practice at the same time to generate either quantitative results or multiple qualitative case studies. Therefore, a single case was selected for this study.

### The Participant

The focal participant in this research was Claudia Mason, the second author of the paper, a non-native Chinese language teacher. Claudia is a European descendant who speaks English as her first language and was born and raised in New Zealand. She studied Chinese as an elective course in addition to her Bachelor’s degree in Psychology and English. After 1 year of Chinese learning (120 h), she won a New Zealand-China government scholarship for a 1-year study abroad programme at Peking University in China. After that, she obtained another scholarship to continue her study of Chinese in Beijing. However, due to the outbreak of COVID-19, Claudia returned to New Zealand and enrolled in a postgraduate programme in Chinese at a university. At the time the study was carried out, she was 23.

During her postgraduate study, Claudia was employed as a tutor to teach the same beginner Chinese course that she had taken as an undergraduate student. Claudia applied for the position along with other candidates and underwent a multi-step evaluation process, which included submitting a lesson plan, giving a teaching demonstration, and participating in an interview, and was successful in securing the position. In her first semester of teaching, Claudia taught 4 days a week for 12 weeks, 1 h per day, independently in the classroom. She also held office hours for 1 h per week and attended group planning meetings every week with six native-speaker tutors. She gave feedback on students’ homework, administered tests, and marked test papers. Her teaching performance was officially evaluated by the university, in line with the evaluations conducted for all tutors. The entire course had 181 students that year and was divided into seven streams taught by seven tutors, including Claudia.

### Data Collection and Analysis

Data collection followed the principles of ITM and much qualitative case study research related to identity, which requires researchers to use multiple sources and types of data to depict teacher identities in the psychological, behavioural, and rationale domains (Dugas, 2021). The first author, a university Chinese lecturer and teacher educator, became Claudia’s supervisor. First, Claudia was asked to produce her autobiographical life story before the teaching began, describing her past experiences, intersectionality with race, gender, upbringing, and first contact with Chinese people and culture. This life story reconstructed her sense of self and her experiences over time and across narratives. Second, Claudia offered to keep a daily teaching reflection journal recording the key events in her professional work during the 12 weeks of teaching. Throughout the semester, Claudia tracked her progress as a new teacher and mapped out her past experiences. Third, in Week 5, she carried out a self-study scrutinising her own professional practices in the classroom. This action research component provided a detailed analysis of Claudia’s debut as a novice teacher. For that purpose, she video-recorded one class she taught in order to later critically examine and reflect on her own behaviours, transcribed the recording, and wrote a report for her postgraduate degree.

Overall, the process of collecting this data was participant-led, rather than being determined exclusively by the researcher (the first author), in order to allow for as much self-exploration as possible by Claudia as a novice teacher (Trent, 2010). As a new teacher, however, Claudia had actively engaged in consultation, discussion and negotiation process to enrich her understanding of the analytical framework (ITM) applied in this study. Furthermore, the study employed a qualitative approach and prioritised ethical reflexivity in order to be aware of the roles and responsibilities of both authors and to ensure the rights of the case study participant (Claudia), including her right to be a co-author of this article, while using her personal data and experiences to answer the research question (Greene & Park, 2021).

Data analysis follows a coding method with a priori goals. Saldaña (2013) noted that pre-established codes “are essential to studies about identity” (p. 62), as identity can only be understood within specific theoretical paradigms. To this end, this study used pre-determined categories identified by ITM, the overarching conceptual framework of this study, which as noted earlier includes psychological, behavioural, and relational domains. Under these broader categories, the next step of coding used a thematic analysis approach to generate codes based on repeated words, phrases, and statements that Claudia often used to further break down the three main categories and gain a more nuanced understanding of her identity as a NNST of Chinese in New Zealand. Finally, by analysing the existing literature, conceptual frameworks, and data, the data analysis identified nine sub-categories of NNST identities.

## Findings

### The Psychological Domain

Data analysis revealed that Claudia attempted to integrate and create coherence among her new teaching experiences and her ongoing social life narratives both across contexts and over time. Claudia’s most frequent identity negotiations were found to be related to her efforts in connecting her unplanned teaching experience, as described below, with her past life experience.

*An accidental teacher* In all sources of data, Claudia expressed an awareness that becoming a Chinese language teacher began as a coincidence rather than a planned career path. Through the (re)construction of her life narrative, she demonstrated a subjective, intrapersonal experience of sameness and continuity in her character. It was not until she composed her life story that she began to realise that her Chinese learning journey no longer appeared to be spontaneous or fragmented—from being a casual learner to a good learner, from a good learner to a scholarship winner for a 1-year Chinese study trip, and finally a Chinese language teacher in the classroom—but rather, that it all made sense to her. In her life story, she wrote:

In my last year of undergraduate study, I decided to take a beginner Chinese language course as an elective paper. This turned into studying Mandarin for a year in Beijing, and now after three years, I have returned back to New Zealand, teaching the exact same course that I once attended as a student.

It can be seen that taking this teaching job became a valuable opportunity for her to unify her past experiences and fragmented identities. She wrote in her reflection journals that “I can conclusively say that analysing my journey of becoming a teacher has been exponentially more helpful to my identity development than simply being a teacher.” Researching her own identity construction as a NNST while engaged in teaching was a chance for her to make sense of her past experiences as a whole person.

*An impostor* One interesting topic that warrants special attention in understanding learner-turned-teachers is Claudia’s use of the term “Impostor Syndrome” (Bernat, 2008) to describe her strong feelings of self-doubt about her capability to teach Chinese. These feelings were particularly strong at the beginning of her teaching practice and persisted throughout most of her reflection journals.

In the beginning of 2021, I accepted the job of teaching a Mandarin language class to undergraduate students at my New Zealand university. This was my first time ever teaching Mandarin, so this new venture obviously carried a new set of trepidations and personal concerns. When I started teaching, a minor thought briefly crossed my mind: Will I be able to do it well? Am I really qualified for this job?

As can be seen, Claudia was inevitably influenced by the native-speakerism that permeates second language teaching (Vallente, 2020). It is clear that the prevalence of native-speakerism, the overemphasis on overcoming linguistic challenges, and a competency-oriented, neoliberal higher education context have created a community where NNSTs might not feel comfortable teaching or might not be accepted as legitimate members or practitioners.

*An adventurer* Another psychological factor that stands out as different from previous cases is the role of personal motivation in shaping the teacher identities. Both Liu (2020) and Zhang and Zhang (2018) noted that learners who became instructors in their studies demonstrated strong motivation and a lifelong commitment to an ongoing process of learning Chinese. They stated that their Chinese learner participants were successful in their learning due to their long-held and deeply rooted interest in Chinese culture. However, Claudia emphasised that her interest in Chinese learning had been fairly recent, and becoming a teacher was accidental—because an opportunity had presented itself. She was also aware that she was brought up in a monocultural white Anglophone community in New Zealand and acknowledged that the hegemonic monolingual English environment did not encourage a deeper commitment to (Asian) language learning (Wang, 2020).

My upbringing in New Zealand was fairly bland, having moved from one white community to another. I was not exposed to many other cultures or languages growing up. Thus, the English language took a hegemonic position within my predominantly white world.

It can be seen that Claudia’s NNST identity manifests itself more as an “adventurer” (Gergen, 1991) rather than a long-term, passionate Sinophone seeking membership in the Chinese teaching community. She expressed discomfort as she grappled with the discourse of teachers as successful (native-like) and motivated learners, and the meaning of this current teaching experience as a discontinuity in her life narrative to date of self-as-student. However, Claudia highlighted that her experience demonstrates that student-turned-teacher success, especially at “such a late stage in life”, can serve as encouragement for those eager to try learning other languages but are hesitant to start at in early or later adulthood. She also found that her experience could send a positive message to many more learners of Chinese by showing that they could picture themselves as teachers in a non-English language classroom by being adventurers rather than authorities.

### The Behavioural Domain

Claudia’s autobiographical data shows that she took actions to prepare herself as a teacher before she entered the classroom. Indeed, her identity as a teacher emerged when she was invited to apply for the teaching position. Upon reflection, Claudia believed that her motivation to “muster up the courage” to apply for and eventually accept the position was due to confidence she gained from study abroad experience, which is described in this section.

*Impact of study abroad* Claudia identified her 1-year study abroad experience as the most critical period of her life that changed her behaviours. This is similar to the six NNSTs in Liu and Wang’s (2018) study, who all took formal Chinese coursework at the tertiary level before studying abroad. This study abroad experience was an important rite of passage that enhanced their language skills and intercultural competence (Grabowski et al., 2017). However, the data shows that Claudia did not see study abroad primarily as a means of improving her proficiency or cultural understanding. Instead, she acknowledged that the change of space had become a catalyst for her to take risks and change her behaviour as an individual (Kayi-Aydar, 2015). In her personal narrative she wrote:

One critical period of my life that evidently influenced my journey through learning Mandarin was travelling to China in 2019... A critical moment during my study abroad was when I competed in the international student speech competition at Peking University. This experience embodied the emergence of a different side to me.

In her life story, Claudia described her self-identity as a “good student,” a “nerd,” and a “self-disciplined Christian girl.” She had always been a quiet and obedient student, never seeking to be put in the spotlight, although the excerpt that follows provides a counter-example. She explained that her motivation to apply for this teaching position was due to her view of Chinese learning as a space where she could “reinvent” herself and try new things in her life, taking on challenges that gave her new life experiences, just as participation in the speech competition in China had done.

I was chosen as the class representative for the speech competition, I think perhaps because […] I appeared quite extroverted and outspoken during class time. […] Looking back, I think I grew into myself in China because it was a space where I could reinvent myself. [Performing in the speech competition] was a concrete accomplishment that stands out as something definitive, almost tangible, in my Mandarin learning journey.

*Impact of learner identity* Her past experience as a language learner and student made Claudia particularly concerned about “making students comfortable.” Her analysis of her own classroom discourse prompted her to moderate her teaching practices as a teacher. For example, she found that she was “overexplaining concepts” because she felt she had the responsibility to help her students in areas that used to challenge her when she was “sitting over there” as a student. While NNSTs are believed to be better at explaining difficult concepts by using students’ first and more familiar language (English in this case) (Wang, 2015), they can also spend too much time on nuanced differences or underestimate students’ ability to work out complex learning tasks on their own. That may be particularly true for novice teachers such as Claudia. As the following excerpt shows, she realised that being a teacher also required her to be able to “lead” instead of “feed”:

Something that was immediately noted [by me] was the ratio of teacher-to-student talk-time. I had often over-explained concepts which the students should have already been familiar with. One of the factors that possibly exacerbated this problem is the desire not to “embarrass” or “frustrate” the students too much. After reflecting on this transcript, I became more aware of the need to allow students to come to their own conclusions, guiding them where necessary.

Reflecting on one’s teaching practices can lead to changes in teacher identity and beliefs, as demonstrated by Tripp and Rich (2012) through their analysis of videotapes of their own teaching. Claudia’s case also reminds us of the importance of recognising that teacher identity is not only constructed through ideas, but also through actions and behaviours in the classroom and feedback from students.

*Impact of social identity* Another topic pertinent to Claudia’s behavioural changes was the influence of her social identity. Evidence of this can be found in her own analysis of her journal entries and her actual teacher talk in the classroom. For example, she realised that she used indirect pragmatics to make suggestions:

I remember during my job interview [for the teaching position] I used a lot of possibility modal verbs such as “we would use this [in these particular situations]” in order to soften a harsh or direct way of framing an answer or statement. One of the interviewers said this kind of language was very Kiwi of me: it’s not necessarily that I’m unsure of the answer, it’s that we use softer language so as not to appear domineering or forceful.

The use of weak modal verbs and the subjunctive mood is the most common thread throughout the transcript of Claudia’s instructional language. For instance, phrases such as “if I were to ask [student]…,” “if we were to answer this question,” and “if I were to say […] in Chinese…” were consistently used in her sentences throughout the entirety of the lesson. This could certainly be attributed to a sense of “Kiwi-ness” as a linguistic feature of New Zealand culture and ways of using language. According to Trent (2010), the use of modality in teacher narratives reveals how teachers identify themselves. Claudia’s personal use of modality reveals a combination of a distinct Kiwi identity, as well as attempts at moderating her behaviour as a novice teacher.

### The Relational Domain

Relational identity directly relates to how Claudia was recognized by others as a certain kind of person (Gee, 2001). Dugas (2021) notes that when new teachers assume a teacher identity, they are particularly sensitive to how others treat them when their teacher identities are revealed or enacted.

*Being more aware of the influences of personal attributes* A new NNST teaching a foreign language such as Chinese in their own country can lead to awkward encounters as they work to build up a teacher–student relationship. One memorable case for Claudia involved being mistaken for a student rather than a teacher due to her young age and ethnic (European) appearance. She described the episode in the following excerpt:

As I was walking into the classroom building, a student passed by and asked me if I was in her biology class. I answered, “no, sorry, I do Chinese.” On reflection, I found this interaction interesting as her assumption that I was a fellow student reinforced some of my fears that I wasn’t a ‘proper’ teacher and that my authority as a teacher wouldn’t be taken seriously. I perhaps internalised this assumption and chose to omit that “I taught Chinese” and instead opted to use language that was more vague: “I do Chinese.”

Through her passing interaction with a student, Claudia realised that her teacher identity could be challenged in the relational domain due to her personal attributes. It is clear that the intersectionality of being a young, white, female played a role in how she imagined herself and how she thought others viewed her. This demonstrates that personal attributes such as race, gender, and age can be key factors shaping how a new teacher might be treated by others.

This incident also illustrates that a NNST’s identity may not be readily enacted at the beginning of their professional practice (Jung & Hecht, 2004). This finding confirms that identity is not only a salient psychological aspect of self and self-concept, but also a central construct in building social relationships. In this case, Claudia’s word choice (*doing* vs. *teaching* Chinese) was also an example of behavioural reflections of identity. This example demonstrates the importance of considering the whole person in gaining a more holistic understanding of a new NNST’s identity.

*Overcoming perceived vulnerability* The fear of not being taken seriously by her students appeared in different places throughout Claudia’s narrative data. While some of these concerns were due to her novice teacher identity, others were directly related to her own identity as a NNST, which she perceived as vulnerable and disadvantaged compared to native-speaker teachers. Common ideas that emerged in Claudia’s journal entries included difficulties explaining words or rules that are “intuitive” for native speakers and answering students’ questions for which she did not have immediate answers. In her first few weeks of teaching, her perceived vulnerability centred around not being a native speaker who possessed the “authentic” authority. In her diary, she wrote:

I remember in the first week of teaching Chinese, it was quite nerve-wracking. I was worried that the students would not take me seriously, or would not view my authority as authentic, especially as I look rather young and am not a native speaker of Chinese.

She acknowledged that before becoming a teacher, she was preoccupied with the fear and perceived vulnerability of being a NNST, which was shaped by the influence of pervasive native-speakerism.

*Deconstructing deficit thinking* Claudia’s teaching evaluation was filled with positive comments from students about her teaching, including her ability to explain difficult words with accurate English instruction and her patience in answering students’ inquiries. Her empathetic teaching style was greatly valued as a form of peer learning by the other six native-speaker peers who observed her teaching. The mutual interaction with students and positive feedback from her students and peers helped to boost her confidence as a NNST and challenged her prior perceptions of NNSTs as vulnerable professionals. By the end of her 12 weeks of teaching, she no longer saw her NNST identity through a deficit-oriented perspective, a perspective that her reflections later in the teaching term revealed:

However, over the weeks, I’ve realised that these fears are not necessarily justified. I may have internalised these negative imagined perceptions, such as that I’m not “authentic”, which may have initially impacted my teacher identity. In realising that some of these negative perceptions are my own creation, I can work to reshape how I view myself as a teacher.

Claudia’s reflections show that NNSTs may need support and guidance in overcoming deficit thinking about their identity in the initial stages of teaching. By the end of the course, she believed that she had achieved congruence between her inner life narrative and how she was positioned by others in the process of transitioning from a student teacher or language learner to a teacher.

## Discussion

This study has provided a rich description and insights into the underexplored process through which a non-native speaker of Chinese developed and negotiated her identities as a novice teacher. The findings revealed that a holistic approach to understanding this teacher’s life and work was in line with the current notion of identity and the human desire to reconcile competing identities into a unified self, regardless of time and space. The analysis of Claudia’s data revealed that she struggled with the dichotomy of being both a learner and a teacher, leading to feelings of illegitimacy, inadequacy, or “imposter syndrome”. However, through self-reflection and analysis of her own dialogical narratives, she was able to observe the struggle between the need to appear professional as an all-knowing source of knowledge and the desire to collaborate with students in her ongoing process of becoming a Chinese language teacher in the New Zealand context.

By using the Identity Triangle Model, this study has integrated the various domains of teacher professional identity constructs using multiple sources of data from a language learning context (Vitanova, 2017), which was not the focus of Dugas’s (2021) research. We argue that research on teacher identity in transitional periods should take into account not only immediate behavioural reactions in the classroom, but also the psychological developments and prior perceptions of the teacher, as well as their enacted identities through interactions with others (Dugas, 2021). Additionally, the Identity Triangle Model allows for a more nuanced understanding of NNST identity as a multi-layered, constantly-evolving concept that attempts to unite their life experiences and make sense of their career choice (Vitanova, 2017).

Amongst the nine sub-categories under the three domains of the ITM, it is clear that Claudia viewed herself as an accidental teacher because it was not part of a longer-term plan or any pedagogical training. Claudia’s motivations for becoming a professional in the language sector were not driven by a strong desire for success or the pursuit of financial stability, but rather by a personal desire for growth and the opportunity to reinvent herself in a new cultural context. This finding is supported by recent research that has shown that contemporary Chinese language learners are less motivated by instrumental factors, such as the desire to improve their job prospects (Wang, 2020). It also suggests that traditional views of language learning and teaching, which focus on success and competitiveness, may not be applicable to non-native speakers of a foreign language teaching in Anglophone countries such as New Zealand. In this regard, this study aligns with Norton’s (2000) critique of the second language acquisition canon and advocates for a more decentred view of language teachers, moving away from binary categorisations as “competent or not competent” compared with a native-speaker teacher, “successful or not successful” in their language learning, or “motivated or unmotivated” for a teaching career. Such a shift could allow for a more inclusive environment in which a wider range of learners feel less stressed to consider teaching as a viable career option, even if only on a short-term basis.

## Conclusion

This case study examined the identity negotiations of a European non-native speaker teacher over the course of 12 weeks during her first semester of teaching Mandarin Chinese at a university in New Zealand. Drawing on the Identity Triangle Model as a new conceptual framework, it provides a holistic understanding of the psychological, behavioural, and relational domains that appeared to influence the identity construction process of a learner-turned-teacher. It also has offered us strong evidence that NNST identity is an ongoing and transformative construct that can only be fully grasped through a unified framework of language teacher identity.

Although the study focuses on a single case in the field of Chinese language teaching, its findings have both theoretical and practical implications for teacher education and teacher development. Future research is recommended to adopt a unified framework, such as the ITM, to better understand the identity negotiation process for first-time teachers. This will provide teacher educators with a multi-layered understanding of new teachers’ personal and social experiences and inform the development of sustainable strategies for NNST empowerment, which has been identified by Llurda and Calvet-Terré (2022) as one of the most pressing research agendas. Additionally, it is suggested that teacher recruitment strategies should consider approaching language learners by sharing inspiring and real stories like Claudia’s (Swanson & Mason, 2018). More research like this one is important as research on new teachers’ life stories and identity negotiations can provide valuable insights for language learners (potential language teachers) and inform their understanding of the challenges and solutions they may encounter in their journey to becoming a language teacher.

One limitation of this study is that it is based on a single case over a relatively short duration, with intersecting factors (age, gender, race, training, expertise) likely affecting Claudia’s perspectives and experiences. Additional research involving a larger number of NNSTs across a range of foreign language programs at universities and schools would be beneficial in order to gain generalizable insights about how these factors operate with other novice language teachers. Another limitation is that the Identity Triangle Model, which was used to analyse the experiences of new teachers as they strive to achieve integration and coherence between their teaching experiences and their ongoing life narratives (Dugas, 2021), has not been extensively tested longitudinally or on more experienced teachers. Despite these limitations, it is important to recognise that each individual’s story and interpretation is unique.

At the time the paper was published, Claudia was pursuing a Master’s degree in Speech and Language Pathology with a scholarship in another New Zealand university. Her experience in Chinese learning and teaching had motivated her for a career in the language sector. Although she had aspirations of becoming a teacher of Chinese, limited teaching opportunities were available when she completed her postgraduate study. It is hoped that Claudia’s example will serve as a source of motivation for more language learners to venture into language teaching (Llurda & Calvet-Terré, 2022), despite any trepidations they may have about the perceived level of commitment and competence needed to become a teacher.
